# Myricetin Increases Hepatic Peroxisome Proliferator-Activated Receptor **α** Protein Expression and Decreases Plasma Lipids and Adiposity in Rats

**DOI:** 10.1155/2012/787152

**Published:** 2012-03-08

**Authors:** Chia Ju Chang, Thing-Fong Tzeng, Shorong-Shii Liou, Yuan-Shiun Chang, I-Min Liu

**Affiliations:** ^1^School of Chinese Pharmaceutical Sciences and Chinese Medicine Resources, China Medical University, Taichung, Taiwan; ^2^Department of Internal Medicine, Pao Chien Hospital, Pingtung City, Pingtung County, Taiwan; ^3^Department of Pharmacy & Graduate Institute of Pharmaceutical Technology, Tajen University, Yanpu Township, Pingtung County, Taiwan

## Abstract

The aim of this study was to investigate the antiobesity and antihyperlipidaemic effects of myricetin. Myricetin exhibited a significant concentration-dependent decrease in the intracellular accumulation of triglyceride in 3T3-L1 adipocytes. The high-fat diet (HFD)-fed rats were dosed orally with myricetin or fenofibrate, once daily for eight weeks. Myricetin (300 mg kg^−1^ per day) displayed similar characteristics to fenofibrate (100 mg kg^−1^ per day) in reducing lowered body weight (BW) gain, visceral fat-pad weights and plasma lipid levels of HFD-fed rats. Myricetin also reduced the hepatic triglyceride and cholesterol contents, as well as lowered hepatic lipid droplets accumulation and epididymal adipocyte size in HFD-fed rats. Myricetin and fenofibrate reversed the HFD-induced down-regulation of the hepatic peroxisome proliferator activated receptor (PPAR)**α**. HFD-induced decreases of the hepatic protein level of acyl-CoA oxidase and cytochrome P450 isoform 4A1 were up-regulated by myricetin and fenofibrate. The elevated expressions of hepatic sterol regulatory element binding proteins (SREBPs) of HFD-fed rats were lowered by myricetin and fenofibrate. These results suggest that myricetin suppressed BW gain and body fat accumulation by increasing the fatty acid oxidation, which was likely mediated via up-regulation of PPAR**α** and down-regulation of SREBP expressions in the liver of HFD-fed rats.

## 1. Introduction

Obesity is a common chronic disorder of carbohydrate and fat metabolism which is characterized by excessive fat deposition in adipose tissue and other internal organs such as liver, heart, skeletal muscle, and pancreatic islet [1]. Obesity remains a major global public health issue because of its increasing prevalence, cutting across all sex, age-groups, ethnicity, or race [1]. Obesity alone can induce all symptoms of metabolic syndrome, which is associated with many additional health problems, including increased risk of insulin resistance, nonalcoholic fatty liver, atherosclerosis, degenerative disorders such as dementia, some immune mediated disorders such as asthma, and certain cancers [2]. Pharmacological approaches to weight control have become an overriding priority [3]. Current trends for obesity management involve multiple pharmacological strategies including blocking nutrient absorption, modulating fat metabolism, regulating adipose signals, and modulating the satiety center, however, several serious adverse effects in clinic, including gastrointestinal adverse effect and significant unfavorable effects on cardiovascular system [3]. As a result, much safer therapeutic is necessary.

Currently, there is growing interest in the therapeutic applications of bioflavonoids and other naturally occurring polyphenols for the treatment and prevention of diseases in humans. Myricetin (3, 5, 7, 3′, 4′, 5′-hexahydroxyflavone) is a naturally occurring flavonoid that is commonly found in tea, berries, fruits, vegetables, and medicinal herbs. Myricetin has been shown to possess antioxidative and cytoprotective properties [4, 5]. A therapeutic effect of myricetin in patients with cardiovascular diseases associated with diabetes mellitus has also been reported [4, 5]. Myricetin has also demonstrated the ability to improve glucose utilization, lowering plasma-glucose levels in a type 1 diabetes-like animal model [6]. Furthermore, myricetin displays the characteristics of rosiglitazone, demonstrating improved glucose utilization and ameliorating the impaired insulin-signaling pathway in insulin-resistant rats induced by the high intake of fructose [7]. Although myricetin has been found to reduce hyperglycemia in diabetic rats, possibly through its ability to increase hepatic glycogen synthesis and to normalize hypertriglyceridemia [8], insufficient information is available regarding the effect of myricetin on the regulation of lipid disorders.

Diet-induced obesity in rodents has been used as an animal model to investigate interactions between the environment and genetics. Rats fed a high-fat diet (HFD) become obese and show distinctive visceral adiposity, dyslipidemia, hyperinsulinemia, and hepatic steatosis, which are typically associated with human obesity [9]. Therefore, a rat model of diet-induced obesity can be used to investigate the effects of antiobesity agents. This study investigated the effects of myricetin on body fat and lipid profiles in rats with diet-induced obesity and sought possible mechanisms of action.

## 2. Methods

### 2.1. Cell Culture

3T3-L1 preadipocytes, obtained from Bioresource Collection and Research Center (BCRC 60159) of the Food Industry Research and Development Institute (Hsinchu, Taiwan), were cultured in Dulbecco's Modified Eagle's Medium (DMEM) (GIBCO BRL Life Technologies, Invitrogen Corporation, CA, USA) with 10% fetal bovine serum (FBS) (GIBCO BRL) and antibiotics (100 units mL^−1^ penicillin and 100 *μ*g mL^−1^ streptomycin). When cells were confluent, differentiation was induced by adding 0.5 mmol L^−1^ isobutylmethylxanthine (Sigma-Aldrich Co., St. Louis, MO) and 1 *μ*mol L^−1^ dexamethasone (Sigma-Aldrich Co.) to the cultures. After 2 days cells were allowed to differentiate further by adding 10% FBS and 10 *μ*g mL^−1^ insulin (Sigma-Aldrich Co.) and the medium was changed every 2 days. At day 10, about 80% of cultures were induced to contain triglyceride (TG). Treatments including serum starvation (DMEM only), myricetin (purity ≥97.0%; Sigma-Aldrich Co.), or fenofibrate (purity ≥99.0%; Sigma-Aldrich Co.) were given to differentiated cultures for 8 hours. All other reagents were of analytical grade.

### 2.2. Measurement of the Triglyceride Content

 Oil Red O (Sigma-Aldrich Co.) at 0.2% in isopropanol (Sigma-Aldrich Co.) was mixed with water (3 : 2, v v^−1^) and filtered. Experimental cultured cells were washed with PBS, fixed by paraformaldehyde (4% in PBS, (Sigma-Aldrich Co.) for 5 minutes, incubated with filtered Oil Red O for 30 minutes, and washed twice with PBS. The stained TG was extracted by isopropanol and its quantity was measured at 490 nm absorbance [10].

### 2.3. Animal Models and Treatment Protocols

 Male Wistar rats aged 8 weeks were obtained from the National Laboratory Animal Center (Taipei, Taiwan). They were maintained in a temperature-controlled room (25 ± 1°C) and kept on a 12 : 12 light-dark cycle (lights on at 06:00 h) in our animal center. Food and water were available *ad libitum*. Regular rat diet (RCD) (11 kcal% fat number LM-485, Teklad, Madison, WI) was used as the maintenance and control diet. A purified ingredient HFD with 45 kcal% fat primarily from lard (number D12451, Research Diets, New Brunswick, NJ) was used to induce a rapid increase in body weight (BW) and obesity [11]. The caloric density of the control diet was 3.4 kcal g^−1^; that of the HFD was 4.73 kcal g^−1^, resulting in lower daily food consumption in grams for the rats fed the HFD. All animal procedures were performed according to the Guide for the Care and Use of Laboratory Animals of the National Institutes of Health, as well as the guidelines of the Animal Welfare Act.

After being fed an HFD for 2 weeks, myricetin was dissolved in distilled water for oral gavage administration at the desired doses (75, 150, and 300 mg kg^−1^ per day) in a volume of 2 mL kg^−1^ once a day into the HFD-fed rats. Another group of HFD-fed was treated by oral gavage with 100 mg kg^−1^ per day of fenofibrate for 8 weeks. The dose of fenofibrate was chosen according to other studies with long-term fenofibrate treatment in rats [12]. A further group of HFD-fed and RCD-fed rats were treated similarly but with the same volume of vehicle (distilled water) as was used to dissolve the tested compounds during the same treatment period. Eight animals were used for each experimental group. Water was made available *ad libitum* throughout the experiment.

Eight weeks after the treatment, rats were weighed and blood samples were collected from the lateral tail vein of the animals anesthetized with sodium pentobarbital (30 mg kg^−1^) administered intraperitoneally (i.p.). Samples were centrifuged at 2,000 g for 10 minutes at 4°C. The plasma was then removed and placed into aliquots for the respective analytical determinations. The liver and visceral and subcutaneous white adipose tissues (WAT) were removed after blood was collected, rinsed with a physiological saline solution, weighed, and immediately stored at −70°C.

### 2.4. Biochemical Parameter Analysis

 The diagnostic kits for determinations for plasma glucose (Cat. No. 10009582), total cholesterol (TC; Cat. No. 10007640) and triglyceride (TG; Cat. No. 10010303) were purchased from Cayman Chemical Company (Michigan, USA). The diagnostic kit for determinations for plasma levels of high-density lipoprotein cholesterol (HDL-C) was purchased from Bio-Quant Diagnostics (CA, USA; Cat. No. BQ 019CR), low-density lipoprotein cholesterol (LDL-C) was calculated by using Friedewald's equation [13]. Plasma level of free fatty acids (FFAs) was determined by the FFAs quantification kit obtained from Abcam plc. (MA, USA; Cat. No. ab65341). All samples were analyzed in triplicate. Atherogenic index (AI) and coronary risk index (CRI) were calculated as follows: LDL-C/HDL-C and TC/HDL-C [14, 15], respectively.

### 2.5. Extraction of Hepatic Lipids

 After removal from the animals, part of the samples of fresh liver was collected for analyzing the lipid contained. Liver (1.25 g) was homogenized with chloroform/methanol (1 : 2, 3.75 mL), and then chloroform (1.25 mL) and distilled water (1.25 mL) were added to the homogenate and mixed well. After centrifugation (1,500 g for 10 min), the lower clear organic phase solution was transferred into a new glass tube and then lyophilized. The lyophilized powder was dissolved in chloroform/methanol (1 : 2) as the hepatic lipid extracts and stored at −20°C for less than 3 days [16]. The hepatic cholesterol and triglycerides in the lipid extracts were analyzed with the diagnostic kits which were used in the plasma analysis.

### 2.6. Hepatic Pathological Evaluation

 Small pieces of hepatic tissues taken from experimental animals were fixed in 10% neutral formalin, alcohol-dehydrated, paraffin-embedded, and sectioned to a mean thickness of 4 *μ*m. The histological examination by the aforementioned conventional methods was evaluated for the index of diabetic-induced necrosis by assessing the morphological changes with hematoxylin and eosin (H&E) stain. Liver biopsy was scored according to the criteria as follows [17]: grade 0: no steatosis, normal liver; grade 1: <25% of hepatocytes affected; grade 2: 26–50% of hepatocytes affected; grade 3: 51–75% of hepatocytes affected; and grade 4: >76% of hepatocytes affected.

### 2.7. Adipocyte Pathological Evaluation

 Histological photograph of adipose tissue was analyzed based on the paraffin method using a light microscope. Fresh tissues were fixed immediately in Bouin's solution for 6–12 hours and then fixed tissue was washed under running water. After being dehydrated through different grades of alcohol, the tissues were embedded in paraffin block at 60°C. Eight *μ*m sections were cut and mounted on glass slides coated with an egg albumin and then the paraffin was removed with xylen and alcohol. The glass slides were stained with H&E. After being dehydrated and cleared by alcohol and xylen, the glass slides were mounted in Canada Balsam. Photomicrographs were taken with a Zeiss Axiolab light microscope equipped with a Nikon Microflex HFX microscope camera. The size of epididymal adipocyte was calculated by Image-Pro Plus 7.0 (Media Cybernetics, MD, USA).

### 2.8. Preparation of Hepatic Fractions

 To prepare nuclear fractions, hepatic tissue was homogenized with ice-cold lysis buffer containing 5 mmol L^−1^ Tris-HCl (pH 7.5), 2 mmol L^−1^ MgCl_2_, 15 mmol L^−1^ CaCl_2_, and 1.5 mol L^−1^ sucrose, and then 0.1 mol L^−1^ dithiothreitol (DTT) and protease inhibitor cocktail were added. After centrifugation (10,500 ×g for 20 min at 4°C), the pellet was suspended with extraction buffer containing 20 mmol L^−1^ 2-[4-(2-hydroxyethyl)-1-piperazinyl]ethanesulfonic acid (pH 7.9), 1.5 mmol L^−1^ MgCl_2_, 0.42 mol L^−1^ NaCl, 0.2 mmol L^−1^ EDTA, and 25% (v v^−1^) glycerol, and then 0.1 mol L^−1^ DTT and protease inhibitor cocktail were added. The mixture was placed on ice for 30 min. The nuclear fraction was prepared by centrifugation at 20,500 xg for 5 min at 4°C. The postnuclear fraction was extracted from the liver of each mouse as described here in after. In brief, hepatic tissue was homogenized with ice-cold lysis buffer (pH 7.4) containing 137 mmol L^−1^ NaCl, 20 mmol L^−1^ Tris-HCl, 1% Tween 20, 10% glycerol, 1 mmol L^−1^ phenylmethylsulfonyl fluoride (PMSF), and protease inhibitor mixture DMSO solution. The homogenate was then centrifuged at 2,000 *×*g for 10 min at 4°C. The protein concentration of each fraction was determined using a commercial kit (Bio-Rad Laboratories, Hercules, CA, USA).

### 2.9. Western Blot Analyses

 For the determination of peroxisome proliferator-activated receptor (PPAR), sterol regulatory element binding protein- (SREBP-) 1, and SREBP-2, 30 mg protein of each nuclear fraction was electrophoresed through 8% sodium dodecylsulfate polyacrylamide gel (SDS-PAGE). Separated proteins were electrophoretically transferred to a nitrocellulose membrane, blocked with 5% (w v^−1^) skim milk solution for 1 h, and then incubated with primary antibodies to PPAR*α* (Santa Cruz Biotechnology, Inc., CA, USA; Cat. No. sc-1985), SREBP-1 (Santa Cruz Biotechnology, Inc.; Cat. No. sc-367), SREBP-2 (Santa Cruz Biotechnology, Inc.; Cat. No. sc-5603), and *β*-actin (Santa Cruz Biotechnology, Inc.; Cat. No. sc-130656), respectively, overnight at 4°C. After the blots were washed, they were incubated with goat anti-rabbit and/or goat anti-mouse IgG HRP-conjugated secondary antibody for 1.5 h at room temperature. Also, 30 mg protein of each postnuclear fraction for acyl-CoA oxidase (ACO; Santa Cruz Biotechnology, Inc.; Cat. No. sc-98499) and cytochrome P450 isoform 4A1 (CYP4A1; Santa Cruz Biotechnology, Inc.; Cat. No. sc-53248) was electrophoresed through 10% SDS-PAGE. Each antigen-antibody complex was visualized using ECL Western Blotting Detection Reagents and detected by chemiluminescence with LAS-1000 plus (Fujifilm, Tokyo, Japan). Band densities were determined using ATTO Densitograph Software (ATTO Corporation, Tokyo, Japan) and quantified as the ratio to *β*-actin. The mean value for samples from the vehicle-treated RCD-fed group on each immunoblot, expressed in densitometry units, was adjusted to a value of 1.0. All experimental sample values were then expressed relative to this adjusted mean value.

### 2.10. Statistical Analysis

 All data represented as the mean ± SEM. Statistical differences among groups were determined by using two-way repeated-measures ANOVA. The Dunnett range post hoc comparisons were used to determine the source of significant differences where appropriate. A *P* value <.05 was considered statistically significant.

## 3. Results

### 3.1. Effects on Triglyceride (TG) Content in 3T3-L1 Adipocytes

**Figure 1:**
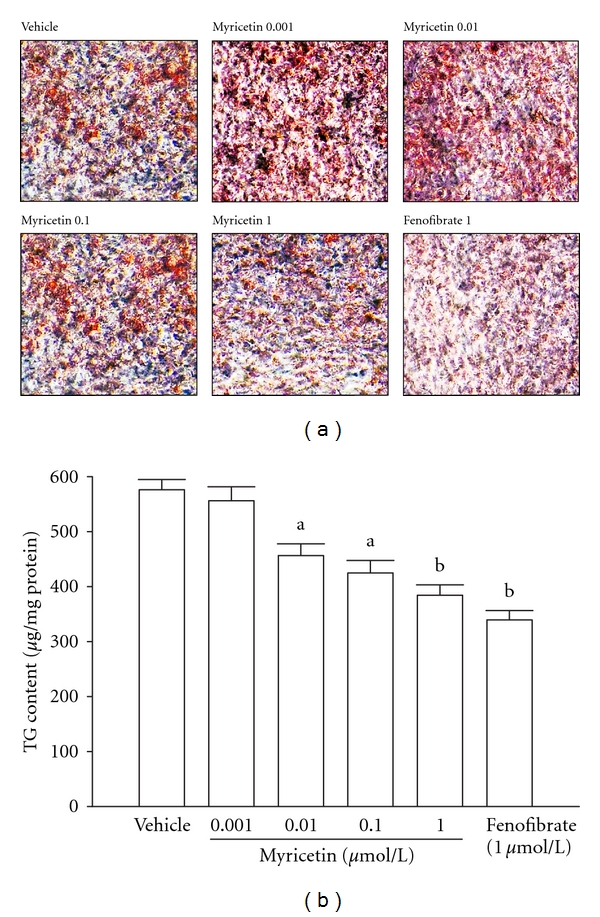
Effect of myricetin or fenofibrate on triglyceride (TG) content in 3T3-L1 adipocytes. (a) Microscopic (40x magnification) images of oil red O-stained adipocytes. Photomicrographs are of tissues isolated from vehicle-treated 3T3-L1 adipocytes (vehicle), myricetin- (0.001 *μ*mol L^−1^) treated adipocytes (myricetin 0.001), myricetin- (0.01 *μ*mol L^−1^) treated adipocytes (myricetin 0.01), myricetin- (0.1 *μ*mol L^−1^) treated adipocytes (myricetin 0.1), myricetin (1.0 *μ*mol L^−1^)-treated adipocytes (myricetin 1.0), or fenofibrate- (1.0 *μ*mol L^−1^) treated adipocytes (fenofibrate 1.0). The vehicle (distilled water) used to dissolve the tested medications was given at the same volume. (b) Quantification of oil red O staining. The experiments were performed in at least 4 replicates per treatment. Values were expressed as mean with SEM (*n* = 4 per group) in each column. ^a^ *P* < .05 and ^b^ *P* < .01 compared to the values of vehicle-treated group.

The TG content of differentiated 3T3-L1 adipocytes treated with myricetin was measured. It exhibited a significant dose-dependent decrease in the intracellular accumulation of TG in 3T3-L1 adipocytes; the most significant effect (over 30% TG reduction) was observed in myricetin treatment at 1 *μ*mol L^−1^ (Figure 1). Fenofibrate (1 *μ*mol L^−1^) caused a decrease in the TG content of differentiated 3T3-L1 adipocytes by 41% (Figure 1).

### 3.2. Effects on Body Weight (BW) and Food Intake

 All the measurements were done after 8 weeks of treatment. The BW of myricetin-treated rats was significantly lower than that of rats in the HFD group (Table 1). The moderate (150 mg kg^−1^ per day) and high doses (300 mg kg^−1^ per day) of myricetin significantly suppressed BW gain; similar results were seen in rats treated with fenofibrate (100 mg kg^−1^ per day; Table 1). No significant differences were observed in daily food intake among all the groups during whole experimental period, despite the water intake became slightly higher in vehicle-treated HFD-fed group as compared to the others (Table 1).

### 3.3. Effects on Fat Pad Weight

The weights of epididymal WAT, perirenal WAT, mesenteric WAT, and inguinal WAT were decreased by 22.2%, 25.1%, 24.4%, and 28.1%, respectively, in myricetin-treated HFD-fed rats compared with their vehicle-treated counterparts (Table 2). The increase in epididymal, perirenal, mesenteric, and inguinal fat pads weight in HFD-fed rats was also lower in fenofibrate-treated group compared with the vehicle-treated counterparts.

### 3.4. Effects on Plasma Lipids

The HFD caused the elevation of plasma TC, TG, and LDL-C concentrations in rats. The moderate (150 mg kg^−1^ per day) and high doses (300 mg kg^−1^ per day) of myricetin significantly decreased the level of plasma TC (23.8% and 31.4% reduction, resp.) compared with vehicle-treated, HFD-fed rats (Table 3). All doses of myricetin decreased the level of plasma TG in HFD-fed rats (Table 3). The low (75 mg kg^−1^ per day), moderate (150 mg kg^−1^ per day), and high doses (300 mg kg^−1^ per day) of myricetin significantly reduced plasma levels of LDL-C (17.5%, 36.8%, and 51.9% reductions, resp.) (Table 3). Plasma TC, TG, and LDL-C concentrations were significantly lower by 41.1%, 51.2%, and 60.1%, respectively, in fenofibrate (100 mg kg^−1^ per day) treated, HFD-fed rats compared with vehicle-treated, HFD-fed rats (Table 3).

Plasma level of HDL-C in HFD-fed rats was lower to 57.8% of that in the RCD-fed group (Table 3). Lower plasma level of HDL-C in HFD-fed rats receiving myricetin (300 mg kg^−1^ per day) or fenofibrate- (100 mg kg^−1^ per day) treatment was elevated to nearly that of RCD-fed group.

The plasma FFAs were significantly higher in HFD-fed rats receiving vehicle (Table 3). The plasma FFA was lower by 39.3% in myricetin- (300 mg kg^−1^ per day) treated HFD-fed rats as compared with the vehicle-treated counterparts (Table 3). FFA concentrations in HFD-fed rats were significantly lower by 46.4% in fenofibrate- (100 mg kg^−1^ per day) treated, HFD-fed rats compared with vehicle-treated, HFD-fed rats (Table 3).

Fenofibrate treatment (100 mg kg^−1^ per day) arrested the elevation of AI and CRI in HFD-fed rats (Table 3). Myricetin treatment also caused a significant (*P* < .05) and dose-related reduction in the atherogenic and coronary artery risk indices in the HFD-fed rats when compared to the values recorded for the vehicle-treated counterparts (Table 3).

### 3.5. Effects on Hepatic Lipids

 The hepatic TC level was significantly higher in HFD-fed rats than that in the RCD-fed group; those were lower by 34.6% in the myricetin- (300 mg kg^−1^ per day) treated group (Table 3). HFD-fed rats receiving treatment with myricetin (300 mg kg^−1^ per day) also showed significantly lower values of hepatic TG to 65.7% as compared with the vehicle-treated counterparts (Table 3). Hepatic TC and TG levels were significantly lower by 40.3% and 41.6%, respectively, in fenofibrate (100 mg kg^−1^ per day) treated rats compared with vehicle-treated, HFD-fed rats (Table 3).

### 3.6. Morphological Changes in Hepatocytes

**Figure 2:**
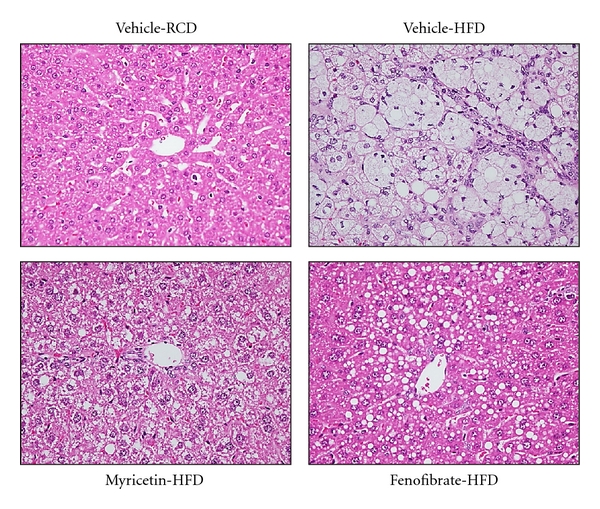
Histopathological findings in livers of HFD-fed rats receiving 8-week treatment with myricetin or fenofibrate. Rats not receiving any treatment were given the same volume of vehicle (distilled water) used to dissolve the test medications. Photomicrographs are of tissues isolated from vehicle-treated RCD-fed rats (vehicle-RCD), vehicle-treated HFD-fed rats (vehicle-HFD), myricetin- (300 mg kg^−1^ per day) treated HFD-fed rats (myricetin-HFD), or fenofibrate- (100 mg kg^−1^ per day) treated HFD-fed rats (fenofibrate-HFD). Photomicrographs were taken at a magnification of ×400.

 HFD-fed rats showed considerable hepatic lipid accumulation compared with that in RCD-fed group (Figure 2). The extent of hepatic lipid accumulation in fenofibrate-treated HFD-fed rats was similar to those in RCD-fed rats (Figure 2). HFD-fed rats receiving treatment with myricetin at the daily dose of 300 mg kg^−1^ showed considerably lower hepatic lipid accumulation than in their vehicle-treated counterparts (Figure 2). The pathological grading of hepatic steatosis in HFD-fed rats was 3.2 ± 0.4, which was reduced to 1.8 ± 0.6 and 1.2 ± 0.3 after receiving treatment with myricetin (300 mg kg^−1^ per day) or fenofibrate, respectively.

### 3.7. Morphological Changes in Epididymal Adipocytes

**Figure 3:**
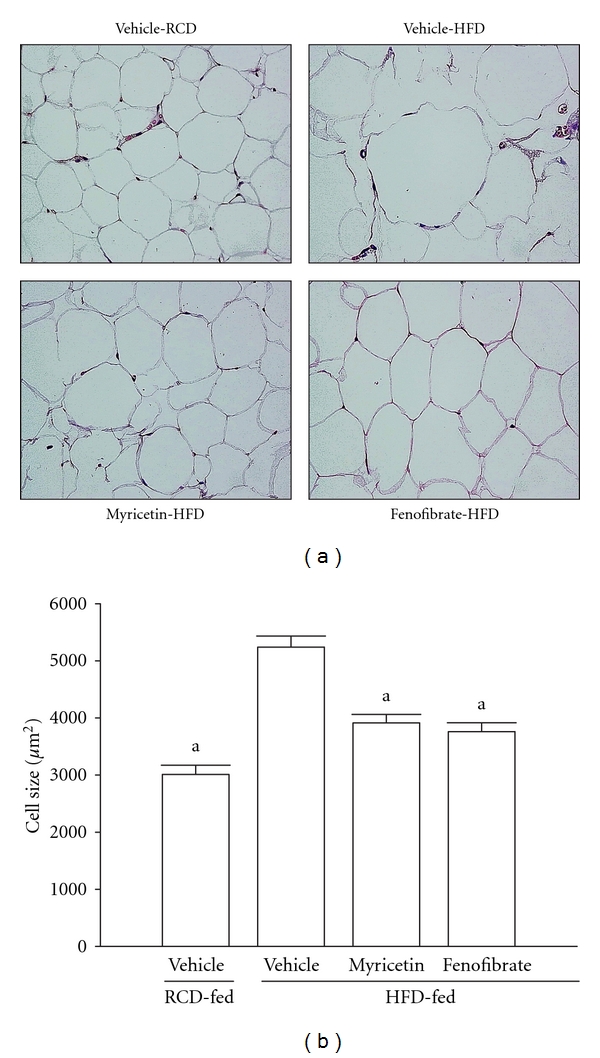
Histopathological findings in epididymal white adipose tissue of HFD-fed rats receiving 8-week treatment with myricetin or fenofibrate. Rats not receiving any treatment were given the same volume of vehicle (distilled water) used to dissolve the test medications. (a) Photomicrographs were taken at a magnification of ×400. Photomicrographs are of tissues isolated from vehicle-treated RCD-fed rats (vehicle-RCD), vehicle-treated HFD-fed rats (vehicle-HFD), myricetin- (300 mg kg^−1^ per day) treated HFD-fed rats (myricetin-HFD), or fenofibrate- (100 mg kg^−1^ per day) treated HFD-fed rats (fenofibrate-HFD). (b) The size of adipocytes in a fixed area (1,000,000 *μ*m^2^) was measured. All values are expressed as mean with SEM (*n* = 5 per group) in each column. ^a^ *P* < .05 compared to the values of vehicle-treated HFD-fed group.

The histological appearance of epididymal adipocyte was irregular in HFD-fed rats compared to RCD-fed group; this morphological change did not appear in HFD-fed groups after fenofibrate treatment (Figure 3). The histological appearance of epididymal adipocyte was more regular in HFD-fed rats treated with myricetin (300 mg kg^−1^ per day). In addition, the sizes of epididymal adipocytes were significantly bigger in HFD-fed group compared to RCD-fed group (Figure 3). The average size of epididymal adipocytes was approximately lower by 25.6% and 32.4%, respectively, in myricetin- (300 mg kg^−1^ per day) or fenofibrate- (100 mg kg^−1^ per day) treated HFD-fed rats as compared with the vehicle-treated counterparts (Figure 3).

### 3.8. Protein Expressions of PPAR*α*, ACO, CYP4A, and SREBPs in Hepatic Tissues

**Figure 4:**
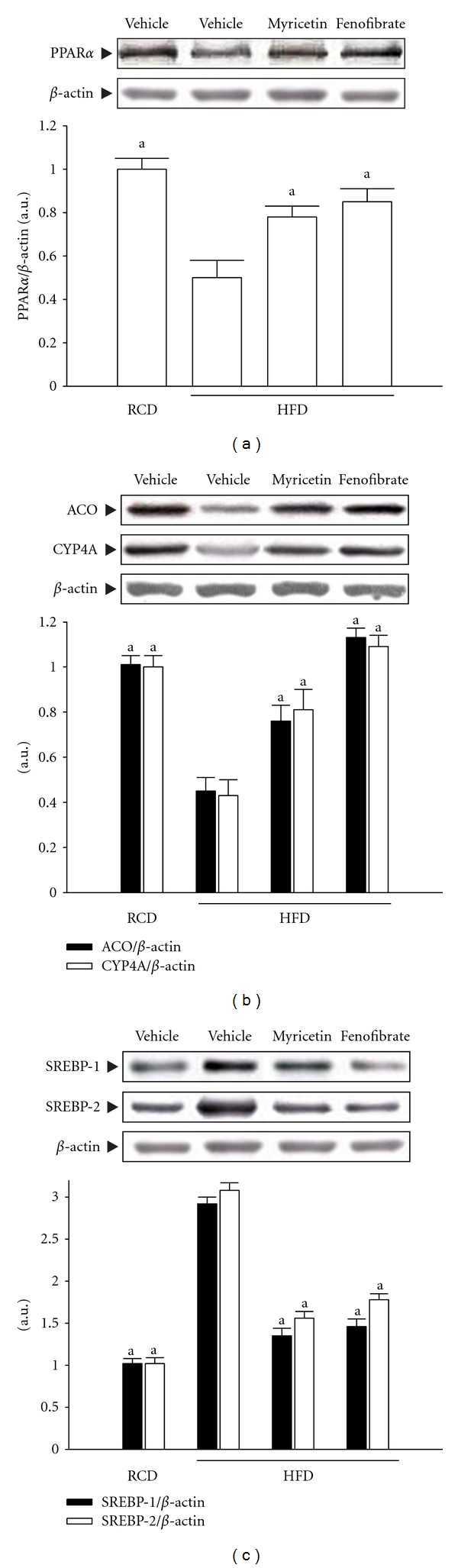
Representative immunoblots of protein expression of (a) PPAR*α*, (b)ACO and CYP4A, and (c) SREBPs in livers of HFD-fed rats receiving 8-week treatment with myricetin (300 mg kg^−1^ per day) or fenofibrate (100 mg kg^−1^ per day). Rats not receiving any treatment were given the same volume of vehicle (distilled water) used to dissolve the test medications. Similar results were obtained with an additional 4 replications. Quantification of protein levels was expressed as mean with SEM (*n* = 5 per group) in each column. ^a^ *P* < .05 compared to the values of vehicle-treated HFD-fed rats.

The hepatic PPAR*α* protein expression of HFD-fed rats was lower than those of the RCD-fed group but significantly elevated by myricetin (300 mg kg^−1^ per day) treatment (Figure 4(a)). In addition, the protein expressions of hepatic ACO and CYP4A in HFD-fed rats were markedly lower than that of RCD-fed group but significantly elevated in myricetin- (300 mg kg^−1^ per day) treated HFD-fed rats (Figure 4(b)). Similar results were seen in rats treated with fenofibrate (100 mg kg^−1^ per day).

The protein expressions of hepatic SREBP-1 and SREBP-2 in HFD-fed rats were significantly higher than those of RCD-fed group, respectively. The HFD-fed rats treated with fenofibrate (100 mg kg^−1^ per day) had hepatic SREBP-1 and SREBP-2 protein expression levels that were 48.6% and 40.2%, respectively, lower than those in vehicle-treated counterpart (Figure 4(c)). Hepatic SREBP-1 and SREBP-2 protein expression levels in HFD-fed rats after myricetin (300 mg kg^−1^ per day) treatment were decreased by 52.3% and 47.3% relative to expression levels in vehicle-treated HFD-fed rats (Figure 4(c)).

## 4. Discussion

Adipocytes play an important role in lipid homeostasis and energy balance by relating to TG storage and free fatty acids release [18]. The antiobesity effect therefore could be represented by the suppression of TG formation in 3T3-L1 adipocytes. Antiobesity effect of myricetin in the 3T3-L1 cell model was measured in our study. We found that myricetin treatment reduced TG content up to 30% at the concentration of 1 *μ*mol L^−1^, which was similar to the effect produced by fenofibrate at same concentration. PPAR*α* is a member of the family of nuclear transcription factors that act as lipid sensors and regulate lipid metabolism [19]. A PPAR*α* activator, fenofibrate, is known to promote fatty acid oxidation and to lower circulating lipids and has been used as a hypolipidemic drug [20].

Several studies have examined the effects of fenofibrate on daily food intake, body weight, and lipid profile in rodent models of obesity [21]. Fenofibrate was applied as the reference drug in this study to investigate the effects of myricetin on body fat and lipid profiles in rats with diet-induced obesity. In the results of this study, long-term HFD feeding resulted in obesity, which was associated with increased BW and fat mass with the development of hyperlipidemia. Similar to fenofibrate treatment, we found that administration of myricetin (300 mg kg^−1^ per day) significantly suppressed the increase in BW in dietary obese rats after 8 weeks. This inhibition did not depend on decreased food or energy intake because all rats in all experimental groups received an equal diet. The BW loss was accompanied by depletion of body fat stores since treatment with myricetin also significantly reduced the weight of the visceral and subcutaneous WAT compared with that of the vehicle-treated HFD-fed group. Excessive growth of adipose tissue results in obesity which includes two growth mechanisms: hyperplastic (cell number increase) and hypertrophic (cell size increase) [22]. The histological appearance of WAT in HFD-group supplemented with fenofibrate or myricetin was more regular and showed similar adipocyte size to that of RCD-group. This suggests that myricetin suppresses HFD-induced adipose tissue mass and BW gain and may inhibit lipid accumulation in adipose tissue in particular.

Obesity, especially abdominal obesity, has an association with dyslipidemia characterized by increasing TG and decreasing HDL-C concentrations [23]. Compelling evidence, from meta-analysis of a number of clinical studies on a large aggregate of patients, has established an increased level of TG as an independent risk factor for cardiovascular disease [24]. TG is involved in the ectopic accumulation of lipid stores in the liver and is associated with a number of diseases such as metabolic syndrome and type 2 diabetes. High TC levels increase the risk of developing coronary heart disease and high levels of LDL-C are also a risk factor for coronary heart disease, while high HDL-C is helpful in transporting excess cholesterol to the liver for excretion in the bile [25]. As a result, HDL-C levels are inversely related to this risk [26]. The present study demonstrated that rats fed an HFD showed a significant increase in plasma TC, TG and LDL-C levels. However, levels of plasma HDL-C in HFD-fed rats were decreased compared with the RCD-group. Similar to fenofibrate treatment, the oral administration of myricetin significantly lowered plasma TC, TG, and LDL-C levels in rats with HFD-induced obesity. Thus, myricetin may be beneficial for treating patients with hypercholesterolemia and hypertriglyceridemia.

The effect of myricetin treatment on the atherogenic and coronary artery risk indices is also notable. The ratio of total cholesterol to HDL-C (also known as the atherogenic index) and the ratio of LDL-C to HDL-C (equally known as coronary artery index) are strong and reliable indicators of whether or not cholesterol is deposited into tissues or metabolized and excreted [27]. In this present study, results showed that treatment with myricetin or fenofibrate caused profound reductions in the atherogenic and coronary indices in experimental hyperlipidaemic rats, which strongly suggested that myricetin has therapeutic potentials in the management of obesity, hyperlipidaemia, and in the prevention of atherogenic cardiovascular diseases.

Due to the ability of myricetin to reduce serum levels of TG and TC, as well as adipose tissue mass and BW gain, which are similar to the function of PPAR*α* activation, we hypothesized that the function of myricetin is related the regulation of hepatic expression of PPAR*α* target genes involved in lipid metabolism. The PPAR*α* is lipid-activated transcription factor that plays a pivotal role in the transcription regulation of genes involved in lipid catabolism and lipoprotein metabolism. In hepatocytes and other tissues (e.g., heart), natural long chain fatty acid (ligand) activated PPAR*α* binds to peroxisome proliferators response element of DNA and increases the transcription of genes encoding enzymes involved in fatty acid oxidation and lipoprotein metabolism [28]. The outcome is an increase in hepatic fatty acid oxidation and ketogenesis, decreased tissue levels of lipids, and protection against lipotoxicity. It was found that myricetin-treated HFD-fed rats had significantly higher hepatic PPAR*α* protein, which was as effective as that produced by fenofibrate. It appears that myricetin transcriptionally increases PPAR*α* target enzymes in the livers, which decreases the intracellular levels of fatty acids available for TG synthesis; this in turn suppresses plasma TG levels and accumulation of fat [29]. Discovery of myricetin as PPAR*α* activator may offer promise of a novel class antiobesity candidate.

Although reductions in the lipogenic activity and in dietary lipid absorption have been suggested as causes of the reduced liver lipid content, an increased capacity of peroxisomal *β*-oxidation (ACO) and microsomal *ω*-oxidation (CYP4A) of fatty acids could also be contributing factors [28]. Elevated hepatic protein levels of ACO and CYP4A imply an enhanced fatty acid oxidation in peroxisomes and microsomes of myricetin-treated HFD-fed rats. These results support the contention that myricetin, by directly or indirectly activating PPAR*α*, can upregulate the expression of PPAR*α* downstream genes, which may lead to enhanced hepatic fatty acid oxidation and reduced TG content.

The sterol regulatory element binding proteins (SREBPs) are a family of three basic helix-loop-helix leucine zipper transcription factors (SREBP-1a, -1c, and -2) that have been identified as transacting factors involved in the maintenance of intracellular cholesterol homeostasis, the control of fatty acid metabolism, and the differentiation of adipocytes [30]. The SREBP-2 isoform activates genes of the cholesterogenic pathway, whereas the SREBP-1 isoforms are more active in regulating the synthesis of fatty acids [31]. It has been documented that SREBPs were the upregulated genes related to fatty acid synthase and cholesterol levels [32]. The elevated expressions of SREBP-1 and SREBP-2 in HFD-fed rats were significantly decreased by the myricetin (300 mg kg^−1^ per day) treatment. These results suggest that myricetin has an ameliorating effect on dyslipidemia through the impaired hepatic SREBPs as well.

In conclusion, the results of this study showed that myricetin suppressed BW gain and body fat accumulation by increasing the fatty acid oxidation, which was likely mediated via upregulation of PPAR*α* and downregulation of SREBP expressions in the liver of HFD-fed rats. We suggest that myricetin may prevent or improve obesity by modulating lipid metabolism and preventing metabolic syndrome as a representative, lifestyle-related cluster of diseases caused by an excessively HFD.

##  Conflicts of Interests

The authors declare that they have no conflict of interests.

## Figures and Tables

**Table 1:** Changes on the body weight (BW) and food and water intake in HFD-fed rats receiving 8-weeks treatment with myricetin or fenofibrate.

Variable (s)	RCD-fed	HFD-fed				
	Vehicle	Vehicle	Myricetin (mg kg^−1^ per day)			Fenofibrate
			75	150	300	(100 mg kg^−1^ per day)
Initial BW (g rat^−1^)	172.5 ± 7.7	173.4 ± 6.9	171.4 ± 7.1	170.3 ± 7.8	173.6 ± 6.5	172.3 ± 6.7
BW gain (g rat^−1^)	17.8 ± 6.2^d^	50.7 ± 7.0^b^	41.4 ± 6.7^b^	30.7 ± 6.9^a, c^	23.6 ± 5.3^d^	19.3 ± 5.5^d^
Food intake (g rat^−1^ per day)	19.3 ± 5.9	20.3 ± 6.7	19.8 ± 6.5	20.7 ± 6.6	20.3 ± 7.8	19.9 ± 7.1
Water intake (mL rat^−1^ per day)	67.2 ± 8.1^c^	88.3 ± 7.4^a^	82.4 ± 9.2^a^	80.8 ± 9.7^a^	78.1 ± 7.9^c^	76.7 ± 8.9^c^

Myricetin or fenofibrate was dissolved in distilled water for oral administration at the desired doses in a volume of 2 mL kg^−1^ once a day into HFD-fed rats. The vehicle (distilled water) used to dissolve the tested medications was given at the same volume. All data represented as the mean ± SEM. ^a^ *P* <  .05 and ^b^ *P* < .01 compared to the values of vehicle-treated RCD-fed rats in each group, respectively. ^c^ *P* <  .05 and ^d^ *P* <  .01 compared to the values of vehicle-treated HFD-fed rats in each group, respectively.

**Table 2:** Changes in the fat-pad weight in HFD-fed rats receiving 8-weeks treatment with myricetin or fenofibrate.

Variable (s)	RCD-fed	HFD-fed				
	Vehicle	Vehicle	Myricetin (mg kg^−1^ per day)			Fenofibrate
			75	150	300	(100 mg kg^−1^ per day)
Epididymal WAT (mg 100 g BW^−1^)	285.4 ± 12.7^d^	396.8 ± 15.6^b^	368.1 ± 16.1^a^	338.5 ± 22.8^a, c^	308.4 ± 19.2^a, c^	293.9 ± 14.6^d^
Perirenal WAT (mg 100 g BW)	162.5 ± 11.6^d^	247.9 ± 12.1^b^	228.6 ± 13.5	198.9 ± 13.9^a, c^	185.3 ± 12.1^c^	170.2 ± 11.7^d^
Mesenteric WAT (mg 100 g BW)	122.1 ± 8.8^c^	180.2 ± 11.5^a^	171.5 ± 13.1^a^	156.2 ± 10.3^a^	136.9 ± 8.7^c^	125.6 ± 9.2^c^
Inguinal WAT (mg 100 g BW)	138.0 ± 10.3^c^	210.2 ± 12.8^a^	194.3 ± 11.8^a^	170.6 ± 11.0^a, c^	151.1 ± 9.2^c^	142.2 ± 10.5^c^

Myricetin or fenofibrate was dissolved in distilled water for oral administration at the desired doses in a volume of 2 mL kg^−1^ once a day into HFD-fed rats. The vehicle (distilled water) used to dissolve the tested medications was given at the same volume. All data represented as the mean ± SEM. ^a^ *P* <  .05 and ^b^ *P* < .01 compared to the values of vehicle-treated RCD-fed rats in each group, respectively. ^c^ *P* <  .05 and ^d^ *P* <  .01 compared to the values of vehicle-treated HFD-fed rats in each group, respectively.

**Table 3:** Changes in the plasma and hepatic lipids, atherogenic index (AI), and coronary artery index (CRI) in HFD-fed rats receiving 8-week treatment with myricetin or fenofibrate.

Variable (s)	RCD-fed	HFD-fed				
	vehicle	Vehicle	Myricetin (mg kg^−1^ per day)			Fenofibrate
			75	150	300	(100 mg kg^−1^ per day)
Plasma TC (mg dL^−1^)	71.4 ± 6.7^d^	143.2 ± 11.9^b^	128.4 ± 9.5^b^	109.6 ± 7.7^a, c^	98.3 ± 7.3^a, c^	84.2 ± 8.0^d^
Plasma TG (mg dL^−1^)	55.7 ± 5.9^d^	134.5 ± 5.7^b^	116.4 ± 6.1^b^	99.8 ± 4.7^b, c^	84.3 ± 4.3^b, c^	65.6 ± 4.7^d^
Plasma LDL-C (mg dL^−1^)	31.2 ± 3.3^d^	103.2 ± 3.9^b^	84.9 ± 5.3^b,c^	66.2 ± 4.7^b,c^	49.5 ± 4.1^a, d^	40.4 ± 4.3^d^
Plasma HDL-C (mg dL^−1^)	45.6 ± 3.1^d^	26.4 ± 3.2^b^	30.5 ± 3.5^b^	37.1 ± 2.9^a, c^	41.7 ± 3.1^d^	43.6 ± 4.4^d^
Plasma FFAs (mg dL^−1^)	27.6 ± 2.7^d^	63.8 ± 4.1^b^	56.9 ± 4.3^b^	48.5 ± 3.8^b, c^	38.7 ± 3.2^a, d^	34.2 ± 3.5^d^
Hepatic TC (mg g^−1^)	2.6 ± 0.8^d^	5.2 ± 0.7^b^	4.6 ± 0.5^b^	4.3 ± 0.4^a^	3.4 ± 0.3^c^	3.1 ± 0.5^c^
Hepatic TG (mg g^−1^)	9.0 ± 1.1^d^	19.0 ± 1.2^b^	17.8 ± 1.4^b^	17.2 ± 1.1^a^	12.5 ± 1.2^c^	11.1 ± 0.8^c^
AI	0.7 ± 0.2^d^	3.9 ± 0.2^b^	2.8 ± 0.1^b^	1.8 ± 0.3^a, c^	1.2 ± 0.2^a, d^	0.9 ± 0.3^d^
CRI	1.6 ± 0.2^d^	5.5 ± 0.6^b^	4.2 ± 0.5^b^	2.9 ± 0.4^a, c^	2.2 ± 0.3^d^	1.9 ± 0.4^d^

Myricetin or fenofibrate was dissolved in distilled water for oral administration at the desired doses in a volume of 2 mL kg^−1^ once a day into HFD-fed rats. The vehicle (distilled water) used to dissolve the tested medications was given at the same volume. All data represented as the mean ± SEM. ^a^ *P* < .05 and ^b^ *P* < .01 compared to the values of vehicle-treated RCD-fed rats in each group, respectively. ^c^ *P* < .05 and ^d^ *P* < .01 compared to the values of vehicle-treated HFD-fed rats in each group, respectively.
